# Personal protection equipment: Preliminary evidence of effectiveness from a three-phase simulation program

**DOI:** 10.1177/17571774231208118

**Published:** 2023-10-18

**Authors:** Ghazwan Altabbaa, Corrinne Pidhorney, Tanya Beran, Joseph Kim, Donna Ledgerwood, Michèle Cowan, Elizabeth Oddone Paolucci

**Affiliations:** 70401 University of Calgary Cumming School of Medicine, Calgary, AB, Canada

**Keywords:** Personal protection equipment, experiential learning, simulation, infection prevention and control

## Abstract

**Background:**

Healthcare providers carry the occupational risk of being exposed to pathogens. Personal Protection Equipment (PPE) requires proficiency whenever used. Yet, evidence shows significant errors and variation in competency when applying PPE.

**Objective:**

In this study, we developed a three-phase intervention to promote PPE proficiency.

**Methods:**

Education and assessment of participants’ PPE knowledge and skills occurred at a large academic university in Western Canada. Participants first completed an online module; second, they experienced one-on-one coaching and deliberate practice with infection control professionals; and third, participants managed a COVID-19 clinical simulation scenario. The measured outcomes include a 15-item pre- and post-knowledge test and a pre- and post-skills assessment of donning and doffing behaviors. These behaviors were observed from video recordings and were assessed using two standardized checklists.

**Results:**

Knowledge and donning/doffing post-test scores (11.73, 0.95, and 0.96, respectively) were significantly higher after completing all three phases of the educational intervention, *p* < .001.

**Conclusions:**

An online module alone is insufficient for PPE knowledge and skill development. Rather, a module followed by practice and simulation allows learners to gain proficiency.

## Background

Healthcare providers are at a risk of being exposed to pathogens, resulting in the transmission or acquisition of illness (Huttunen and Syrjänen, 2014). These risks may become magnified during times of emerging pathogens or pandemics where the likelihood of exposure to a patient’s infectious substances is high. In fact, such risks have been documented repeatedly over the last two decades and during the recent COVID-19 pandemic (Chou et al., 2022; Kilmarx et al., 2014; Summary of probable SARS cases with onset of illness from 1 November 2002 to 31 July 2003). In 2002–2003, 21% (i.e., 1,706 of 8,096) of the Severe Acute Respiratory Syndrome (SARS) cases were healthcare providers, contributing to a 9.6% fatality rate (summary of probable SARS cases with onset of illness from 1 November 2002 to 31 July 2003). Similarly, surveillance data of the 2014 West Africa Ebola Virus Disease showed that the Ebola incidence was 100-fold higher among healthcare providers than in the general population and for those who were more likely to have had contact with a live Ebola patient or ill person in the 30 days previous to symptoms (Kilmarx et al., 2014). Additionally, worldwide epidemiological updates continue to reveal an increased risk of COVID-19 infection risk among healthcare providers (Chou et al., 2022).

Infection prevention and control (IPC) measures are critical in protecting healthcare providers especially when workforce capacity is a crucial consideration. The recent global pandemic has highlighted the importance of preserving adequate healthcare workforce capacity. Various considerations including improved infection control programs, adequate training, and provision of PPE have been proposed (Bourgeault et al., 2020; Patel et al., 2021). System-level interventions that offer protection should integrate effectively with other interventions at the individual level (1910.1030 - Bloodborne pathogens. | Occupational Safety and Health Administration). These include administrative, engineering, and personal controls. The appropriate use of Personal Protective Equipment (PPE) by individual healthcare providers should meet performance standards (Protecting Healthcare Personnel | HAI | CDC 2021 Oct 21) to assure adequate protection against pathogen exposure. However, these performance standards are not easily achieved (Verbeek et al., 2020). A 2020 systematic review found that the probability of contamination, compliance rate, and satisfaction with the use of PPE depended on multiple factors, including design elements, number of covered parts, and types of PPE (Verbeek et al., 2020). Furthermore, in an evaluative study of PPE use, performance outcomes revealed high rates of self-contamination (28–44%), as well as protocol deviations during donning (27–50%) and doffing (67–100%) (Kwon et al., 2017). Several studies found performance gaps in PPE sequence, removal, use of correct equipment, and missed steps (Casanova et al., 2012; Hajar et al., 2019). These findings suggest sub-standard performance and inconsistencies in the correct and safe use of PPE among healthcare workers.

While evidence of educational interventions for improving PPE compliance or reducing errors is mixed (Verbeek et al., 2020), PPE performance is somewhat higher when interactive training methods (e.g., simulation, computer simulation, and face-to-face training) than traditional approaches (e.g., lectures and watching videos) are used (Ward, 2011; Hung et al., 2015; Curtis et al., 2018; Pokrajac et al., 2020). Traditional instructional design based exclusively on lectures or videos lacks the immersive and authentic experience of healthcare contexts where PPE performance standards must be achieved. Within real practice environments, the application of performance standards of the PPE procedure is challenged by multiple potential failure points that inherently exist at the healthcare system (e.g., design, setup of equipment, and space) and individual healthcare provider levels (e.g., competing cognitive tasks, distractions, and produced heat/sweating from wearing PPE). Overcoming and identifying these limitations in achieving PPE performance standards and integrating IPC principles demand designing educational interventions that lead to transformative and collaborative learning experiences. Indeed, high-fidelity simulation is one unique teaching model where real-world interactions, relationships, and cognitive aspects of human and system elements can be simulated within a safe learning and engaging environment. Here, high-fidelity simulation model presents a constructivist teaching and learning strategy by combining the pedagogical, cognitive, and social presence in a safe and collaborative learning environment (Garrison et al., 1999). Such an environment promotes active engagement and deliberate practice. Simulation educators can present learners with a well-defined task (i.e., PPE proficiency) and provide feedback during multiple and varied opportunities for deliberate practice to gradually refine and improve performance to close the gap between the actual and desired level (Ericsson, 2008). Hence, innovations in the design of PPE equipment and knowledge acquisition, via active and engaging educational interventions such as simulation, should reflect the complex and dynamic workplace realities.

This study aimed to assess the effects of a blended experiential educational intervention on PPE proficiency and IPC principles. While this intervention was deployed early in the pandemic to address COVID-19 as a high-consequence infectious disease (HCID), several of the knowledge and skills elements emphasized standard infection prevention principles (SICP) (e.g., hand hygiene and point of care assessment) and additional precautions based on pathogen suspected (i.e., transmission-based precautions). Our primary research question was: Do physicians-in-practice demonstrate improved knowledge and performance in PPE donning and doffing technical skills following completion of a three-step curriculum consisting of an online module, one-on-one coaching, and simulation of a complex clinical scenario?

## Methods

### Study design and setting

A prospective observational design was used in this study to collect pre- and post-assessments from participant learners. While such study designs are known to have limitations (Knapp, 2016), we have avoided creating comparison groups for practical and urgent reasons to provide much-needed training and protection for our healthcare volunteers. The study was approved by the University of Calgary Conjoint Health Research Ethics Board (CHREB). All data were collected at the Advanced Technical Skills Simulation Laboratory (https://cumming.ucalgary.ca/atssl) during the period of April to June 2020.

### Participants

Participant learners included independent practicing physicians from one of the largest healthcare systems and research-intensive universities in Western Canada. This group of physicians responded to the Department of Medicine’s recruitment call early in the pandemic to cover clinical shifts on the COVID-19 units. In addition to PPE training, volunteer physicians were required to complete several refresher clinical modules for hospitalized patient care in addition to standard N95 respirator fit testing. This recruitment was aimed solely at fully licensed physicians who could be deployed immediately at the COVID-19 units at various hospital sites. Physicians in training (i.e., residents or medical students) and allied health professionals (i.e., registered nurses) received differing PPE simulation practice opportunities based on recommendations from their own training programs and unit managers and were excluded from the study. In fact, the recruited physicians belonged to various specialties within the Department of Medicine, with some practicing mainly in outpatient settings and having less exposure to or recent training in PPE standards.

The existing research suggests poor baseline PPE practices of healthcare workers in both simulation (contamination range, 42.5%–50.3) and real hospital (contamination, 90%) settings (Phan et al., 2019; Tomas et al., 2015). To determine how many participants were required to detect statistically significant improvements in our study, an a priori sample size calculation using G*Power 3.1 (Faul et al., 2009; Kang, 2021) was conducted. With a moderate effect size of 0.50, power of 0.90, and α =0.05 for a two-tailed, dependent measures t-test, 44 participants were needed.

### The educational intervention

**Figure 1.**
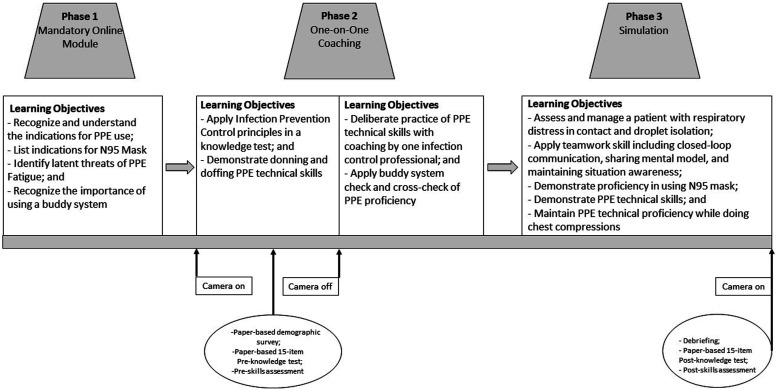
Flow chart of the educational intervention, including the data collection time points.

The educational intervention focused on available protocols and guidelines in the use of PPE for hospitalized patients requiring isolation (Services AH Personal Protective Equipment, Dec 2021). Additionally, principles of infection prevention control were emphasized to help learners recognize and understand the correct type of isolation for different disease presentations (e.g., cough and fever, skin rash and fever, and wounds) (Services AH Resource Manuals, Dec 2021). To address the complexity and multifaceted areas of PPE knowledge and technical skills, the curriculum was divided in sequential phases to allow learners to (i) engage with the content, (ii) assess acquired learning, (iii) receive direct feedback, and (iv) demonstrate skills via a simulated clinical encounter followed by a reflection on patient care and self-exposure (Kirkpatrick and Kirkpatrick, 2016). Infection control specialists and simulation educators used multiple feedback strategies including reflection, coaching, and explicit clarification of performance standards (Nicol and Macfarlane-Dick, 2006). Figure 1 depicts the study’s three phases and learning objectives.

#### Phase 1

During this phase, learners accessed a mandatory online learning session on IPC protocols. This training module was offered and facilitated by the Office of Faculty Development and Performance (OFDP) at the University of Calgary. The curriculum included a basic introduction to modes of pathogen transmission, types and purposes of PPE, basic tenets of IPC, situational awareness regarding clean and dirty spaces, and clinical challenges in applying PPE proficiently.

#### Phase 2

After completion of the online module, learners were scheduled to attend a 2-h simulated lab session. This session occurred within a few weeks of completing phase 1 (mean = 5.48 days, SD = 4.03 days). A researcher greeted participants at the simulation lab and acquired informed consent from those interested in participating in the study. The participation rate was 96%, with only 2 people declining video recording, resulting in a final sample of 44 participants. Those who consented were given a brief survey to complete on demographic characteristics. Then all learners were administered a 15-item IPC knowledge test, which took no more than 10 min to complete. The knowledge test scores were included in the study only from learners who provided consent. These scores served as the pre-test comparison with a later administration of this same test. Next, learners were paired with another learner who had arrived around the same time to practice PPE procedures together. As they began donning their PPE, they were reminded that the camera was being turned on. The video recording of this phase was obtained to serve as a pre-test comparison with a later observation of PPE procedures. Nonconsenting participants were told that if they were in the frame of the camera, their performance would not be included in the study. In the pre-assessment skills assessment phase, participants completed one donning and doffing round of practice without any instruction. Once completed, the camera was turned off and an infection control professional demonstrated the correct approach to the pair of learners. The total duration of this phase was about 1 h.

#### Phase 3

After phase 2, learners were pre-briefed and oriented (10–15 min) to a simulated clinical scenario that required the (i) assessment of a patient with respiratory distress, (ii) application of teamwork skills, and (iii) recognition of the appropriate type of PPE (Appendix A) (services). Each simulation was composed of four people: an infection control professional, a certified simulation educator by the Society of Simulation in Healthcare, and two physician learners. Upon completing a 20-min simulation scenario, learners were debriefed (15 min) by the simulation educator in a standardized manner. Areas of discussion and reflection considered questions such as the type of PPE required, appropriate decontamination measures, and communication procedures under isolation conditions. After the debriefing, the camera was turned on and participants completed a final demonstration of their PPE donning and doffing (i.e., post-procedural skills assessment), as well as the same 15-item paper-based IPC knowledge test (i.e., post-knowledge assessment). Two members of the research team (CP and DL) independently viewed and scored the 44 videos using two standardized checklists (Appendix B), and any discrepancies were discussed to arrive at consensus in scoring. At least one designated infection prevention professional watched all simulation sessions live. Two standardized checklists developed by local infection control professionals (Appendix B) were used to ensure all components were performed and to record PPE adherence, deviations, and donning or doffing difficulties.

### Outcomes

Two types of data were collected (see Figure 1). The 15-item IPC pre- and post-knowledge tests (Appendix C) were completed upon the participants’ arrival (start of phase 2) and then again at the end of the three phases. The questions consisted of a multiple response format that tested knowledge of isolation principles (e.g., What is the main difference in transmission of pathogens via droplet versus airborne route; check all that apply for isolation order when the case involves suspected bacterial meningitis). A higher score indicates a higher number of correct responses for a total maximum score of 15. The second type of data was collected from the video recordings of the pre-test (phase 2) and post-debriefing donning and doffing technique phases (phase 3). Infection control professionals utilized two checklists to assess physician-learner PPE proficiency: (i) Donning Checklist, comprising 12 items measuring how PPE clothing items are correctly put on; and (iii) Doffing Checklist, comprising 17 items measuring the correct removal of PPE clothing. Each checklist item was scored as either 0 (occurred) or 1 (did not occur) and the mean number of items was calculated, with a higher mean indicating more correct behaviors occurring. Infection control professionals created these checklists, which provide evidence of content validity. To ensure calibration in teaching and assessment practices, two of the professionals who taught in this intervention (CP and DL) independently scored 10 randomly selected videos and met to compare and resolve any discrepancies in scoring. Then they proceeded to independently score the remaining videos. The inter-rater reliability was 80% for both pre-test donning and doffing behaviors. However, when taking into consideration chance agreement, the intraclass correlations (ICC) were low to moderate (0.61–0.09) (Koo and Li 2016). Upon closer inspection, we discovered that several rows of data had been misaligned in the spreadsheet from one coder. Thus, the analyses included data from the other coder who confirmed that data entry was correct.

Our primary outcome measures were changes in knowledge scores for individuals based on their pre-test and post-test assessments, as well as changes in behaviors and checklist assessment scores as observed through the donning and doffing of PPE in phases 2 and 3.

### Statistical analyses

All quantitative analyses were conducted using SPSS version 24 (IBM Corp. Released 2016. IBM SPSS Statistics for Windows, Version 24.0. Armonk, NY: IBM Corp.). For the demographic survey items, frequency counts and percentages were calculated for noncontinuous variables. Means and standard deviations were calculated for continuous variables. Dependent measures t-test analyses were conducted for all mean difference within-group comparisons (pre- versus post-test scores). A value of *p* < .05 was considered statistically significant.

## Results

Demographic characteristics about the sample are shown in Table 1. The mean number of years in practice was 12.22 (SD = 9.41). Most participants worked in a variety of areas of medicine and in hospital inpatient/outpatient clinics. They were likely to use PPE in their daily practice and work in situations where PPE was needed. Although 18% (*n* = 8) of our participant physicians reported never using PPE, 52.3% (*n* = 23) indicated they were confident in using PPE.

**Table 1.** Demographic characteristics *N* = 44.

	*n* (%)
Sex	
Male	17 (38.6)
Female	26 (59.1)
Unidentified	1 (2.3)
Area of medicine	
Internal medicine	10 (22.7)
Hematology	9 (20.5)
Other	25 (56.8)
Practice setting	
Hospital inpatient/outpatient	23 (52.3)
Hospital inpatient/outpatient and community	11 (25.0)
Other	10 (22.7)
Frequency of PPE use	
Never	8 (18.2)
Often or more	36 (81.8)
Work in PPE situations	
Yes	34 (77.3)
No	10 (22.7)
Confident in using PPE	
Yes	23 (52.3)
No	5 (11.4)
Neutral	16 (36.4)

As shown in Table 2, participants’ knowledge and technical skills in donning and doffing PPE significantly improved from the pre-test to the post-test. That is, all three scores were higher after completing the three-step curriculum consisting of an online module, one-on-one coaching, and simulation of a complex clinical scenario (represented in the post-test column) than completing only the online learning module (represented in the pre-test column).

**Table 2.** Knowledge and technical skills scores change over time.

	Pre-test *Mean (SD*)	Post-test *Mean (SD*)	*p*	Effect size (*d*)	Pre-test *Mean (SD)* % items correct	Post-test *Mean (SD)* % items correct	
Knowledge test	6.61 (2.20)	11.73 (2.07)	<.001	2.89^a^	44.07 (14.68)	78.18 (13.82)	
Donning skills	0.79 (0.17)	0.95 (0.07)	<.001	0.17^b^	79.32 (17.31)	95.00 (7.31)	
Doffing skills	0.51 (0.22)	0.96 (0.08)	<.001	0.25^b^	50.62 (22.29)	96.45 (8.21)	

^a^effect size in the large range.

^b^effect size in the medium range (Cohen, 1988).

## Discussion

This study obtained preliminary evidence of a simulation educational intervention that enabled learners to practice and experience challenges in the use of PPE. High-fidelity simulation can offer a training environment that is dynamic, complex, and psychologically safe, while still mimicking the challenging realities of the healthcare setting. The curriculum was introduced thoughtfully to allow for an experience that integrates a combination of strategies, including one-on-one coaching, direct feedback, and deliberate practice (Nicol and Macfarlane-Dick, 2006). The goal was to present learners with and guide them through a challenging clinical situation to foster a proper understanding of IPC principles and the application of PPE standards and skills.

The study showed significant improvements in the high range for knowledge gain and in the moderate range for technical skills. Specifically, learners showed these gains *after* completing an online module, thereby suggesting that this sole form of education is insufficient for learners to gain mastery. This finding highlights a critical consideration: PPE education should not rely solely on passive online training. For learners to further expand their understanding and application of PPE knowledge, additional formats such as guided practice and simulation are needed. We are unable to determine which of these improvements were the result of the practice, coaching, discussion, simulation, or debriefing because the assessment was not conducted after each component. It is also possible that improvement occurred because of the multiple feedback discussions that were integrated into the different phases of the education program. These specific mechanisms can be studied in future research.

Central to the strength of the study, is the deliberate insertion of multiple transition events that mimic the dynamic nature of healthcare settings where latent threats can lead to failure points and errors (i.e., moments where performance could be affected due to unperceived challenges). For example, the simulation scenario starts with a patient case of mild respiratory distress and hypoxia. The patient improves to a limited degree by upgrading the oxygen delivery device but is then followed by further decline requiring the consideration of advanced respiratory support. Such inserted transitions are expected to trigger a participant’s demonstration of repeated reassessments of respiratory status, already established PPE protocol, and early anticipation of an aerosol-generating medical procedure while demonstrating simultaneously key teamwork skills such as situational awareness, sharing the mental model, and closed loop communication. Another example is the cardiac arrest transition event. Here, each participant delivered 2 min of chest compressions in real-time while monitoring self and others for any breaches in PPE protocol. In addition, due to the physical demands, the two-minute chest compression phase allowed participants to experience some discomfort (i.e., sweating and heat) while wearing PPE and simultaneously remaining vigilant about aspects of executing high-performance chest compressions. The scenario ended after each participant completed 2 min of chest compressions and transitioned into the doffing procedure and exiting the room. This doffing step was considered one of the most important latent threats leading to a performance failure point. In fact, existing evidence suggests the presence of high rates of doffing errors and a mismatch between self-perceived proficiency and correct use of the PPE (Fogel et al., 2017; Poller et al., 2018). Our study observed similar findings with multiple high-risk breaches in the doffing technique during this phase (e.g., participants touching and removing the N95 mask immediately after finishing chest compressions; participants removing gowns in a hurried and uncontrolled manner). These breaches were addressed by the infection control professional observers utilizing a variety of feedback methods, including demonstration, direct feedback, and coaching. Taken together, this multi-faceted sequential instructional design was focused on replicating clinical stimuli and cues that are typically present in dynamic clinical environments to support attaining the desired learning outcomes which would lead to safe practices for our healthcare providers and their patients.

There were several limitations in this study. First, this curriculum was implemented during the earliest stages of the COVID-19 pandemic. It is possible that the knowledge gaps and uncertainties at that time may have created a heightened sense of perceived risk of exposure from work, community, or both, thereby increasing the motivation to participate and learn. While risk perception is known to be a powerful driver of motivation to gain knowledge on how to protect oneself (Ferrer et al., 2018), it is thought that the observed change in adherence behavior was a natural and necessary response to seeking knowledge on how to manage uncertainty and cope with the evolving unpredictability in healthcare practice. Furthermore, the design of the simulation scenario incorporated many elements of standard infection control precautions (SICP) (e.g., hand hygiene and point of care assessment) which are considered standard practice, while requiring participants to understand the basic mechanisms of transmission with respect to suspected pathogens (i.e., transmission-based precautions). As such, there was a combination of design elements (i.e., HCID, SICP, and TBP) that are generally applicable to clinical situations where one must understand and demonstrate basic principles of infection prevention and control and the use of appropriate PPE. Second, participants were not randomly selected and may not be representative of other physicians in practice. Moreover, there was no comparison group (i.e., participants that did not participate in any of the three education phases). However, we did not want to draw a separate group from the total number of volunteers to form a comparison group because we wanted them all to have the full education program so that they would be as prepared as possible to support the healthcare response for patients with COVID-19. There was a sense of urgency at the time of the study as the pandemic had just been declared, motivating us to promptly conduct this study. Third, there was no follow-up to assess whether the increased PPE proficiency was sustained. However, studies of advanced resuscitation skills training suggest that simulation-based interventions improve skill retention (Au et al., 2019). Therefore, it is recommended that PPE training courses adopt a parallel design approach for greater impact and consider research opportunities to assert if booster sessions are beneficial for enhancing long-term skills retention. Finally, the inter-rater reliability according to the ICC was low to moderate after the initial training. Thus, future research needs to further examine the reliability of scores from these checklists.

For these practices and skills to be maintained over time, these education opportunities must not only be available but also deliberately integrated at the system level. As a result of the above encouraging results, the Internal Medicine Simulation Program at this university in Western Canada continues to offer a translation and application of the training model to rounding residents and medical students on the medical unit at one of the original participating hospital sites during interprofessional simulation sessions that include registered nursing staff who work on the same medical unit. Additionally, the Post Graduate Medical Education (PGME) office currently mandates and supports the completion of all three steps of this training model within 1 week prior to the start of all incoming residents and specialty fellows. Finally, the Office of Faculty Development and Performance (OFDP) at the same university in Western Canada now offers an online PPE certification course that is embedded in the standard educational curriculum offered to all healthcare workers in training and practice.

PPE education should focus on curriculum design formats that mimic the dynamic and complex realities of healthcare settings. This study provides preliminary evidence that the implementation of such a design promotes an integrated understanding of basic IPC principles and PPE proficiency.

## Supplemental Material

Supplemental Material - Personal protection equipment: Preliminary evidence of effectiveness from a three-phase simulation program Click here for additional data file. Supplemental Material for Personal protection equipment: Preliminary evidence of effectiveness from a three-phase simulation program by Ghazwan Altabbaa, Corrinne Pidhorney, Tanya Beran, Joseph Kim, Donna Ledgerwood, Michèle Cowan, and Elizabeth Oddone Paolucci in Journal of Infection Prevention

Supplemental Material - Personal protection equipment: Preliminary evidence of effectiveness from a three-phase simulation program Click here for additional data file. Supplemental Material for Personal protection equipment: Preliminary evidence of effectiveness from a three-phase simulation program by Ghazwan Altabbaa, Corrinne Pidhorney, Tanya Beran, Joseph Kim, Donna Ledgerwood, Michèle Cowan, and Elizabeth Oddone Paolucci in Journal of Infection Prevention

Supplemental Material - Personal protection equipment: Preliminary evidence of effectiveness from a three-phase simulation program Click here for additional data file. Supplemental Material for Personal protection equipment: Preliminary evidence of effectiveness from a three-phase simulation program by Ghazwan Altabbaa, Corrinne Pidhorney, Tanya Beran, Joseph Kim, Donna Ledgerwood, Michèle Cowan, and Elizabeth Oddone Paolucci in Journal of Infection Prevention
