# Supplementary material for: Personal protection equipment: Preliminary evidence of effectiveness from a three-phase simulation program
Source: J Infect Prev. 2023 Oct 18;24(6):244–51. doi: 10.1177/17571774231208118 (PMC10638951; doi:10.1177/17571774231208118)
Supplement: Supplemental Material - Personal protection equipment: Preliminary evidence of effectiveness from a three-phase simulation program [file sj-pdf-1-bji-10.1177_17571774231208118.pdf]

ROCKYVIEW SIMULATION

INTERNAL MEDICINE SIMULATION PROGRAM

Scenario Planning Worksheet

Clinical Scenario	Acute respiratory failure in COVID-19 patient
Programmed scenario?	No
Medical discipline	Inpatient Medical Unit
Target learners	Faculty, Residents
Estimated time of scenario	15 minutes
Estimate time of debriefing	20 minutes

Simulation Team

Voice & control	Nurse	Scenario director	Confederate(s)	RT	Session Evaluator	Debriefing 1	Debriefing 2: Infection Prevention consultant
☒	☐	☒	☒ RN	☐	☐	☒	☒

Learning objectives

General management

1. Assess and manage a patient with respiratory distress.
2. Apply teamwork skills, including closed-loop communication, sharing mental models, and establishing and maintaining situational awareness.
3. Recognize when to call for help.
4. Appreciate the need to establish/discuss goals of care.
5. Demonstrate PPE proficiency during a simulation situation of a respiratory distress case.
6. Recognize cardiac arrest.
7. Each learner is to apply effective chest compression for 2 minutes.

Case specific

1. Recognize the appropriate type of PPE for specific patient care procedures, such as the Use of Aerosol Generating Medical Procedure
   Basic Life Support.
2. Demonstrate proficiency in using N95 respirator.
3. Evaluate the roles of all healthcare providers for the purpose of mitigating unnecessary exposure.
4. Evaluate the need for specific workups and treatments for the purpose of mitigating unnecessary exposure (e.g., EKG, BiPAP, nebulizer).
5. Monitor for breaches of PPE protocol of healthcare team members during patient care assessment and basic life support.
6. Demonstrate buddy-system check and cross-check while donning and doffing.
7. Consider strategies for bringing equipment into the isolation room (e.g., stethoscope, monitoring devices).

The Rockyview General Hospital Internal Medicine Simulation Program © 2015 is an accredited program by the Royal College of Physicians and Surgeons of Canada

	8. Consider strategies for communication between the team inside the isolation room and support personnel outside the room.<br>9. Demonstrate appropriate decontamination procedures for used equipment (e.g., stethoscope, penlight).<br>10. Discuss and demonstrate strategies/next steps for healthcare providers who might have become exposed (e.g., skin/wrists, touching the face, touching mask, open gown).

Brief Summary:

In this scenario, the learners will assess a patient with acute respiratory distress admitted to a medical unit with ILI presentation. The patient develops worsening oxygen demands complicated later with cardiac arrest. Learners must be thoughtful about required workup, immediate use of various oxygen devices, and provide effective chest compression while adhering to PPE protocols.

A summary of optional progress: The scenario director may not introduce the cardiac arrest event if he/she is observing significant PPE breaches. In this situation, the scenario could be paused, give direct feedback on the PPE procedure, and allow some time to understand/correct errors. The scenario could be resumed later (or restarted from the beginning) to continue with rapid cycle debriefing or complete debriefing at the end of the scenario.

Equipment checklist

Personal			
☒	Universal precautions equipment (gloves, mask, gown…), contact and respiratory	☐	
☒	Stethoscope	☐	
☐	Penlight	☐	
☒	Reference material: contact and droplet signs and cognitive aids: entry and exit points	☐	
Diagnostic			
☒	BP cuff	☒	Medical records
☐	Thermometer	☒	Imaging: EKG, labs, Chest x ray
☒	SpO_2 monitor and probe	☐	

The Rockyview General Hospital Internal Medicine Simulation Program © 2015 is an accredited program by the Royal College of Physicians and Surgeons of Canada

☒	ECG monitor/Defib/Pacer	☐	
☒	Lab reports	☐	
☐	Physician orders	☐	
Therapy			
☒	Oxygen source	☒	IV pump
☒	Oxygen delivery devices (nasal cannula, high-flow nasal cannula, non-rebreathing mask, high-flow nebulizer	☐	
☐	Suction device and suction catheter (tonsil tip and flexible)		Oropharyngeal airway and/or nasopharyngeal airway
☒	IV start kit		Blood drawing equipment and tubes
☒	Nebulization delivery device	☐	Arterial blood gas equipment
☐	Crash cart	☐	
☐		☐	
Drugs and fluids			
☐	Pip/Tazo	☐	Metronidazole
☒	Ceftriaxone	☐	
☐	Ciprofloxacin	☐	
☒	NS	☐	
☐	Azithromycin	☐	

The Rockyview General Hospital Internal Medicine Simulation Program © 2015 is an accredited program by the Royal College of Physicians and Surgeons of Canada

ROCKYVIEW SIMULATION
INTERNAL MEDICINE SIMULATION PROGRAM

Preparation of the simulator & environment					
**Monitor Setup**					
Monitor Layout	3 waves monitor				
Parameters					
☒	Primary ECG	☐	CVP	☒	AWRR
☐	Secondary ECG	☒	NBP	☐	CO
☐	Arterial BP	☐	TOF	☒	TPERI
☒	SpO_2 (Pleth)	☐	N_2O	☐	TBLOOD
☐	PAP	☐	O_2	☐	ICP
☐	CO_2	☒	Pulse	☐	AGT

Patient description

ID: Pt is a 48-year male admitted last night from ER to the medical unit.

Case introduction/entry notification:

Unit MD: We are on the inpatient medical unit; you are the MD taking over the care of this patient, I am the MD who was on call last night; I need to discuss/handover of this patient to you.

Pt is 48 years old male admitted last night with ILI symptoms and is currently in contact and droplet isolation.
No PMH, R1 goals of care.
Since admission, he has been stable, and I haven't been paged about him in the last couple of hours. Oxygen saturation has been okay on 3 L NC; BP is stable now on 0.9 NS 125 L/h but requires a couple of litres in ER. Currently, he is on IV ABX. He had EKG, Chest X-ray, and some labs in ER. All swabs, including COVID-19, are still pending.

The general appearance of patient: GCS 15, mild shortness of breath, and coughing intermittently. NC tube on the side of the face (not secured well).

Patient/Voice control/speech/mood: Weak but able to give history and answer questions.

The Rockyview General Hospital Internal Medicine Simulation Program © 2015 is an accredited program by the Royal College of Physicians and Surgeons of Canada

MD/Nurse/confederate instructions:
Provide the handover outside the room with the door closed.
Contact and droplet isolation sign on the door. Donning and doffing visual cognitive aids are posted on the door/wall outside and inside the room.
Provide patient's chart including H/P, work up (Chest x-ray, EKG, labs), and invite the learners to start their donning protocol. Then leave the space. IPC professionals could be present to observe and/or guide through the protocol.

Confederate family member: No family member confederates.

Vital signs:
Vitals in Triage: BP 85/60, HR 130 regular, RR: 32, SpO2 88-89% on RA. Temperature 38.2
Vitals today: 110/85, HR 100, RR: 24, SpO2: 92% nasal cannula 3 L/min Temperature: 37.7
Vitals on monitor NOW when entering the room: HR 125, BP 125/80, RR 28, SpO2: 84% RA (NC off face), Temperature: 37.8

☒Show this information on the monitor before starting the scenario: vitals on the monitor will be shown upon entering the room.
☐Make this information available upon request

Additional information, medical history

☒Show this information in the chart after introducing the case: Provide a full chart but leave it outside the room per protocol for patients in isolation.
☐ Make this information available upon request

Patient data:
Gender: male, age: 48, weight 160 pounds, Height: 5'7"

History of present illness:
The patient starts by telling the team that the NC tubing fell off his face, and he couldn't put it on again properly.
"Pt. is a 48-year-old male with approximately a 2–3-day history of shortness of breath, coughing yellow sputum, feeling warm, weakness and fatigue, body aches, and sore throat. He wasn't eating much over the last several days, felt a bit dizzy at home, and was more breathless when walking to the bathroom; he called the emergency line yesterday and was advised to come to the hospital. His wife drove him to ER. There were no sick contacts, and nobody is sick at home."

The Rockyview General Hospital Internal Medicine Simulation Program © 2015 is an accredited program by the Royal College of Physicians and Surgeons of Canada

Review of systems:
CNS: Some headaches yesterday, no vision changes, no paresthesia.
CVS: Progressive dyspnea over last few days on exertion, no orthopnea, no PND, no palpitations, no edema, no chest pain.
Respiratory: Cough with some yellowish sputum, no hemoptysis.
GI: No nausea vomiting, or diarrhea, and no abdominal pain.
GU: No dysuria, no urine discoloration, decreased urine output, but still urinating.
Skin: No rash.
ID: Feeling warm, no diaphoresis, no chills, positive sore throat, generalized body aches over last few days.

Past medical history: None
Allergies NKDA
Home Medications: Tylenol PRN, coughing syrup.
Current Medications: 0.9 NS at 125 ml/h, Ceftriaxone 1 g 24h, Azithromycin 500 mg q24, Innohep 4500 units qd.
Surgical: None
Social: He works as a schoolteacher and lives with his wife. No smoking, No ETOH but drinks occasionally, no IVDA. No travel history, no sick contacts, no illness in the family.
Goals of Care: R1 (full basic and advanced resuscitation measures).

Physical examination
Vitals in Triage: BP 85/60, HR 130 regular, RR: 32, SpO2 88-89% on RA. Temperature 38.2
Vitals today: 110/85, HR 100, RR: 24, SpO2: 92% nasal cannula 3 L/min Temperature: 37.8
Vitals now on the monitor: HR 125, BP 125/80, RR 28, SpO2: 84% RA (NC off face), Temperature: 37.6
General inspection: Mild respiratory distress, no accessory muscle use, eyes half-closed.
HEENT: No pallor, no icterus, PERRLA, oral cavity: dry mucous membranes.
Neck: JVP 2 cmH2O
Chest: No wheezing, course crackles bilaterally.
CVS: S1 S2 normal, no murmur.
Resp: Inspiratory coarse crackles to the lung bases bilaterally. Decreased breath sounds the lung bases bilaterally.
Abdo: Soft, no tenderness, no organomegaly, no distension, bowel sounds present in all quadrants.
CNS: PEARLA, GCS = 15, no meningismus, reflexes normal.
Derma: No rash; skin feels warm.
Extremities: No mottling, no edema, the pulse is symmetrical and present in all four extremities, no DVT signs.

The Rockyview General Hospital Internal Medicine Simulation Program © 2015 is an accredited program by the Royal College of Physicians and Surgeons of Canada

Proposed flow of correct approach/treatment		
**Events**	**Desired Actions**	**Comments**
Introduction & Handover	- Review medical records outside the isolation room.<br>- Notice the contact and droplet isolation sign.<br>- Proceed with doffing.<br>- Secure a good seal by putting an N95 mask on before entering the contact and droplet isolation room.<br>- Recall the fitted size of the N95 mask.	Educator:<br>- Provide direct feedback and coaching on PPE use if needed.<br>- Encourage buddy check and cross-check of PPE.
Entering the patient's room<br>Vitals on the monitor:<br>HR 125, BP 125/80, RR 28, SpO2: 84% RA (NC off face), Temperature: 37.8<br><br>The patient is coughing and speaking short sentences.<br>A - patent airway<br>B - Bilateral crackles, shortness of breath, increased work of breathing<br>C - Tachycardia	- Make introductions to patients.<br>- Recognize that the NC tube has fallen off.<br>- Recognize and interpret the vital signs monitor showing hypoxia.<br>- Replace the NC with prior oxygen FIO2 (at previous FIO2 of 3 L/M).<br>- Proceed with clinical assessment, including current symptoms and physical examination.<br>- Evaluate response provided oxygen treatment at 3 L/M NC.<br>- Optimize FIO2 by increasing FIO2 via High flow NC (HFNC).<br>- Request fitting size of N95 masks if not already on.<br>- Replace face mask with N95 mask following proper procedure and secure good seal.	Learners:<br>- Anticipate the need for Aerosol Generating Medical Procedures (e.g., HFNC, NRB mask, nebulizers).<br>- Consider having fewer staff in the room. Leave charts (computers) outside the room.<br>- Leave staff outside the room (runner, documentation).<br>- Learners should request their own fitted size of N95 mask.

Transition 1:<br>Vitals on the monitor:<br>HR 135, BP 125/80, RR 32, SpO2: 87% RA (high flow NC), Temperature: 37.8<br>The patient remains dyspneic.	- Recognize that oxygen saturation remains critically low with current oxygen therapy.<br>- Recognize increased respiratory rate and respond to the prompt of increase of work of breathing.<br>- Optimize the FIO2 further via a non-rebreathing mask (NRB)<br>- Request workup for respiratory distress (repeat chest x-ray, ABGs).<br>- Recognize worsening respiratory status (hypoxemic respiratory failure) and chest x-ray findings compared to prior studies.	Learners:<br>- Possible to consider NRB oxygen device immediately and skip the step of using HFNC: vital signs monitor will show initial improvement of oxygen sat to 87% with NRB followed by a decline down to 84% as outlined in transitions.<br>- Consider strategies for communicating outside the room.
Transition 2:<br>Vitals on the monitor:<br>HR 140, BP 125/80, RR 36, SpO2: 84% RA (NRB), Temperature: 37.8<br>Patient remains dyspneic.<br>Eyes half-closed	- Maintain situation awareness and recognize the further decrease in oxygen saturation on NRB therapy.<br>- Recognize increased respiratory rate and respond to the prompt of increase of work of breathing.<br>- Declare the emergency: acute hypoxemic respiratory failure that requires advanced respiratory life support	Learners:<br>- Share mental model.<br>- Call for help (code).<br>- Anticipate high-risk AGMP (bag-valve ventilation, intubation).
Transition 3:<br>Vitals on the monitor:<br>Cardiac arrest<br>Eyes fully closed<br>Patient not responsive<br>No pulse	- Maintain situation awareness and recognize that pt is not responsive.<br>- Check pulse<br>- Declare cardiac arrest situation: no pulse<br>- Call for help (code blue)<br>- Hands on the chest<br>- Apply chest compression<br>- Maintain PPE proficiency and monitor for exposure	Learners:<br>- Leave staff outside room (runner, documentation)<br>- 2 minutes of *real-time* chest compression for each learner<br>Educators:<br>- Provide feedback about rate and depth to achieve effective chest compression<br>- If the learner asks about BVM, intubation, or crash cart: report that it is not available until code blue team members have arrived and completed their donning, including N95 masks<br>- Can provide a CPR board when asked

The Rockyview General Hospital Internal Medicine Simulation Program © 2015 is an accredited program by the Royal College of Physicians and Surgeons of Canada

		- Can provide a step stool when asked.
Transition 4:<br>- The scenario director declares that the Code Blue team has arrived.<br>- Code Blue team is taking over the resuscitation.<br>- Pull the curtain around the mannequin.<br>- Notify learners that they need to exit the space.<br>- Notify learners that they need to proceed with the doffing of PPE.	- Doffing of PPE in adherence to technical standards.<br>- Exiting the room.<br>- Decontamination of used equipment (e.g., stethoscope).	Educators:<br>- Watch for their safety as they step down from the step stool.<br>- Watch for their safety as they move away from the mannequin (e.g., tripping on oxygen or IV lines).<br>- Notify learners that doffing is part of the simulation exercise.<br>- Direct the learners to the doffing cognitive aid inside the room if needed.<br>- Provide direct feedback and/or demonstration if needed.<br>- Monitor for high-risk behaviours leading to exposure (e.g., touching face mask with contaminated gloves, removal of the gown in an uncontrolled/hurried manner).<br>- Provide direct feedback and/or demonstration for decontamination procedures if needed.
