# Supplementary material for: Personal protection equipment: Preliminary evidence of effectiveness from a three-phase simulation program
Source: J Infect Prev. 2023 Oct 18;24(6):244–51. doi: 10.1177/17571774231208118 (PMC10638951; doi:10.1177/17571774231208118)
Supplement: Supplemental Material - Personal protection equipment: Preliminary evidence of effectiveness from a three-phase simulation program [file sj-pdf-2-bji-10.1177_17571774231208118.pdf]

Donning Checklist

1. Preparation

- ☐ Remove extra items on person (rings, watch, unnecessary wrist jewelry etc.)
- ☐ Secure long hair off of face and neck

2. Hand Hygiene

- ☐ Perform hand hygiene using alcohol-based hand rub. If hands look or feel dirty, or patient with diarrhea use soap and water.

3. Gown

- ☐ Insert arms through sleeves
- ☐ Ensure gown covers from neck to knees to wrist
- ☐ Tie at the back of neck
- ☐ Tie at the back of waist

4. Procedure / Surgical Mask

- ☐ Secure ties or elastic bands around head (or ears) so that the mask stays in place
- ☐ Mold the nose bridge band to your nose.
- ☐ Mask completely covers nose and chin and is secure to prevent slippage

5. Eye Protection or face shield (if not integrated into the procedure mask)

- ☐ Place eye protection over the eyes. If using a face shield, place band around the head with foam perpendicular to the forehead.
- ☐ Adjust to fit

6. Gloves

- ☐ Pull cuffs of gloves over the cuffs of the gown

Rationale:

1. Jewelry and extra items can get caught in your PPE, especially on removal, resulting in self-contamination, putting you at risk. Such items can become contaminated as a result of breaches in PPE or unconscious touching/practice and are difficult to clean. Rings or sharp edges can cause tears and breaches in your PPE. Hair can become contaminated through splashing, touching, or being tangled in PPE.
2. When donning PPE, it is important to start off with clean PPE. Performing hand hygiene removes germs that may contaminate your clean PPE.
3. Careful and methodical donning of your PPE is important to ensure proper coverage and movement for your body. Donning begins with your gown to ensure proper coverage around your neck and torso (including your back). Securing the ties prevents the gown from dragging across contaminated objects in the environment and prevents unconscious practices of reaching under your gown to retrieve items on your person (e.g., pens, pagers, phones, resources)
4. Careful donning/securing of the mask ensures that your mouth and nose are properly covered. If your mask is not secure, it may slip and result in you consciously or unconsciously touching it to adjust, thereby risking contamination of your mask and mucous membranes. A poorly fitted mask can result in gaps around your mouth and nose, which defeat the purpose of wearing a mask in the first place.
5. If you are thinking you should protect your respiratory tract, you should also be thinking about protecting your eyes as they are a mucous membrane. Microbes can land on your eyes, and the eyes can become a portal of entry into your body. Eyeglasses do not provide sufficient protection from droplets.
6. Gloves should cover the cuffs of your gown, as this will significantly reduce the risk of contamination of your hands, wrists, and arms during your doffing (removal of your PPE).

Doffing Checklist

INSIDE ROOM
**1. Gloves**<br>☐ Grasp outside edge of glove near the wrist and peel away from the hand, turning the glove inside-out. Hold removed glove in opposite (still gloved) hand<br>☐ Slide an ungloved finger or thumb under the wrist of the remaining glove.<br>☐ Peel the glove off<br>☐ Put gloves in the garbage.
**2. Hand Hygiene**<br>Perform hand hygiene using alcohol-based hand rub. If hands look or feel dirty, use soap and water.
**3. Gown**<br>☐ Carefully unfasten ties. (Necktie first.)<br>☐ Grab the inside of one cuff and pull over the hand gaining control of the end.<br>☐ Grab the middle of the other cuff.<br>☐ Keeping arms level gently pull the gown away from your body turning the gown away from you. Do not roll the gown.<br>☐ Place in the hamper in the patient room or, if disposable, put in the garbage.
4. **Hand Hygiene**<br>☐ Perform hand hygiene using alcohol-based hand rub. If hands look of feel dirty, use soap and water.<br>☐ Exit room. (If door is closed when leaving patient room, ensure to perform hand hygiene again prior to removal of eye protection.)
OUTSIDE ROOM
**5. Eye Protection or Face Shield**<br>☐ Handle only by headband or earpieces.<br>☐ Carefully pull away from face.<br>☐ Place reusable items in appropriate area for cleaning. Put disposable items into the garbage.
**6. Procedure / Surgical Mask**<br>☐ Carefully remove the mask from your face by touching only the ties or elastic bands using four fingers for maximum control. (Start with bottom tie and then remove the top tie.)<br>☐ Put mask into the garbage.
**7. Hand Hygiene**<br>☐ Perform hand hygiene using alcohol-based hand rub. If hands look of feel dirty, use soap and water.
