# Supplementary material for: Personal protection equipment: Preliminary evidence of effectiveness from a three-phase simulation program
Source: J Infect Prev. 2023 Oct 18;24(6):244–51. doi: 10.1177/17571774231208118 (PMC10638951; doi:10.1177/17571774231208118)
Supplement: Supplemental Material - Personal protection equipment: Preliminary evidence of effectiveness from a three-phase simulation program [file sj-pdf-3-bji-10.1177_17571774231208118.pdf]

Infection Prevention and Control (IPAC) Principles

Trainee Level: ○ **Medical Student: Year: _____**

○ **Resident: Year: __________** **DATE:**

○ **Other: ____________________**

○ **1st time taking test**

○ **2nd time taking test**

○ **other**

1. **% of adults in Canadian Hospitals with a hospital acquired infection (HAI) at any given time:**

- ○ 3%
- ○ 22%
- ○ 10%
- ○ 50%

2. **On average, how far do droplets of infectious pathogens travel via vomit, sneeze or cough?**

- ○ 0.5 m
- ○ 3 m
- ○ 2 m
- ○ 6+ m

3. **What is the main difference in transmission of pathogens via droplet vs. airborne route?**

- ○ ambient air pressure
- ○ type of pathogen (bacterial vs. viral)
- ○ particle size
- ○ number of air exchanges per hour

4. **What is the minimum effective rub time for hand hygiene using alcohol-based hand rub?**

- ○ 5 seconds
- ○ 20-30 seconds
- ○ 10-15 seconds
- ○ 1 minute

5. **In the context of the 4 Moments of Hand Hygiene, which of the following is NOT patient environment?**

- ○ patient curtain
- ○ bedrail
- ○ bedside table
- ○ healthcare equipment in the patient room

6. **Suspected Bacterial Meningitis - isolation order (check all that apply)**

- ○ none
- ○ droplet
- ○ contact
- ○ airborne

7. **Suspected Bacterial Meningitis - PPE required (check all that apply)**

- ○ none
- ○ procedure mask with eye protection
- ○ gown and gloves
- ○ N95 respirator

8. **GAS Necrotizing Fasciitis - isolation order (check all that apply)**

- ◯ none
- ◯ contact
- ◯ droplet
- ◯ airborne

9. **GAS Necrotizing Fasciitis - PPE required (check all that apply)**

- ◯ none
- ◯ gown and gloves
- ◯ procedure mask with eye protection
- ◯ N95 respirator

10. **Acute Gastroenteritis (with vomiting) - isolation order (check all that apply)**

- ◯ none
- ◯ contact
- ◯ droplet
- ◯ airborne

11. **Acute Gastroenteritis (with vomiting) - PPE required (check all that apply)**

- ◯ none
- ◯ gown and gloves
- ◯ procedure mask with eye protection
- ◯ N95 respirator

12. **Acute fever, diffuse rash, cough, conjunctivitis - (check all that apply)**

- ◯ none
- ◯ contact
- ◯ droplet
- ◯ airborne

13. **Acute fever, diffuse rash, cough, conjunctivitis - PPE required (check all that apply)**

- ◯ none
- ◯ gown and gloves
- ◯ procedure mask with eye protection
- ◯ N95 respirator

14. **Influenza-like illness (ILI) - isolation order (check all that apply)**

- ◯ none
- ◯ contact
- ◯ droplet
- ◯ airborne

15. **Influenza-like illness (ILI) - PPE required (check all that apply)**

- ◯ none
- ◯ gown and gloves
- ◯ procedure mask with eye protection
- ◯ N95 respirator
